# Desynchronization Strain Patterns and Contractility in Left Bundle Branch Block through Computer Model Simulation

**DOI:** 10.3390/jcdd9020053

**Published:** 2022-02-06

**Authors:** Kimi Owashi, Marion Taconné, Nicolas Courtial, Antoine Simon, Mireille Garreau, Alfredo Hernandez, Erwan Donal, Virginie Le Rolle, Elena Galli

**Affiliations:** INSERM, CHU Rennes, University of Rennes, LTSI–UMR 1099, F-35000 Rennes, France; kimiowashi@gmail.com (K.O.); marion.taconne@univ-rennes1.fr (M.T.); nicolas.courtial@univ-rennes1.fr (N.C.); antoine.simon@univ-rennes1.fr (A.S.); mireille.garreau@univ-rennes1.fr (M.G.); alfredo.hernardes@univ-rennes1.fr (A.H.); erwan.donal@chu-rennes.fr (E.D.); virginie.lerolle@univ-rennes1.fr (V.L.R.)

**Keywords:** cardiac resynchronization therapy, left bundle branch block, left ventricular deformation, personalized computational model, myocardial scar

## Abstract

Left bundle branch block (LBBB) is associated with specific septal-to-lateral wall activation patterns which are strongly influenced by the intrinsic left ventricular (LV) contractility and myocardial scar localization. The objective of this study was to propose a computational-model-based interpretation of the different patterns of LV contraction observed in the case of LBBB and preserved contractility or myocardial scarring. Two-dimensional transthoracic echocardiography was used to obtain LV volumes and deformation patterns in three patients with LBBB: (1) a patient with non-ischemic dilated cardiomyopathy, (2) a patient with antero-septal myocardial scar, and (3) a patient with lateral myocardial scar. Scar was confirmed by the distribution of late gadolinium enhancement with cardiac magnetic resonance imaging (cMRI). Model parameters were evaluated manually to reproduce patient-derived data such as strain curves obtained from echocardiographic apical views. The model was able to reproduce the specific strain patterns observed in patients. A typical septal flash with pre-ejection shortening, rebound stretch, and delayed lateral wall activation was observed in the case of non-ischemic cardiomyopathy. In the case of lateral scar, the contractility of the lateral wall was significantly impaired and septal flash was absent. In the case of septal scar, septal flash and rebound stretch were also present as previously described in the literature. Interestingly, the model was also able to simulate the specific contractile properties of the myocardium, providing an excellent localization of LV scar in ischemic patients. The model was able to simulate the electromechanical delay and specific contractility patterns observed in patients with LBBB of ischemic and non-ischemic etiology. With further improvement and validation, this technique might be a useful tool for the diagnosis and treatment planning of heart failure patients needing CRT.

## 1. Introduction

Cardiac resynchronization therapy (CRT) is an established therapy for patients with systolic heart failure and widened QRS (>130 ms) who remain symptomatic despite optimized medical therapy [1]. Despite this, 30–40% of patients receiving CRT according to current recommendations do not experience significant left ventricular (LV) reverse remodeling after CRT [2]. In recent years, several measurements of LV dyssynchrony have been proposed, but none of them have been shown to significantly improve responders’ rate [1]. This might be because only specific mechanical substrates associated with LV electrical delay are amenable to be responsive to CRT [3]. The identification of these substrates is particularly challenging because it should consider the specific electro-mechanical properties of the myocardium, which are highly patient-specific. In this context, computational modeling might be an efficient tool to integrate cardiac electrical activation, mechanical properties, and hemodynamic conditions in data processing. Indeed, various computational models have been proposed to underline the different physiological mechanisms. Most of them are based on the finite element method [4,5,6], including a complete representation of the geometry, which requires high computational resources and is difficult to personalize. Alternative approaches have been proposed to overcome this computational cost by simplifying the patient anatomy [7,8]. Although these models successfully represented a patient diversity in LV dyssynchrony and proposed keys to understand CRT response, patient specification with real and non-invasive clinical data must be made to propose a personalized medicine.

Various models of the cardiovascular system have been previously developed by our team [9,10]. We proposed the first patient-specific model of the left ventricle that successfully simulates myocardial strains in dilated cardiomyopathy [11]. In this paper, a model of the cardiovascular system was adapted from [12], integrating four main sub-models: (1) the cardiac electrical system, (2) right and left atria, (3) multi-segment representation of the right and the left ventricles, and (4) systemic and pulmonary circulations. The model considered not only the electrical activation delay of each segment, but also the differences in regional contractility, which are known to largely contribute to strain morphology and global cardiac mechanics.

The aim of this study was to propose a model-based interpretation of the different patterns of LV contraction observed in patients with LBBB in case of myocardial scarring and non-ischemic cardiomyopathy.

## 2. Materials and Methods

### 2.1. Experimental Data

#### 2.1.1. Study Population

We prospectively included three CRT candidates with typical LBBB (QRS width > 130 ms) participating in ongoing CRT studies at the Department of Cardiology of the University Hospital of Rennes, France. One patient had non-ischemic dilated cardiomyopathy, one patient had ischemic cardiomyopathy with lateral scar, and one patient had ischemic cardiomyopathy with antero-septal scar. All patients underwent transthoracic echocardiography before CRT implantation. We performed cardiac magnetic resonance imaging (cMRI) with late gadolinium enhancement (LGE) to exclude previous myocardial infarction and to localize myocardial scar when needed.

Table 1 summarizes patients’ clinical characteristics. The study was conducted according to the guidelines of the Declaration of Helsinki and approved by the local Institutional Ethics Committee (French Brittany Regional Ethic Committee validation number: 35RC14-9767).

#### 2.1.2. Echocardiography

All patients underwent standard transthoracic echocardiography using a Vivid E9 or E95 ultrasound system (GE Healthcare, Horten, Norway) equipped with an M5S 3.5-MHz transducer before CRT implantation.

LV volumes were measured according to current recommendations, and LV ejection fraction (LVEF) was calculated using the Simpson biplane method [13].

Two-dimensional grayscale images were acquired in the apical 4-, 3-, and 2-chamber views at a frame rate ≥60 frames/s. The recordings were processed offline using an acoustic-tracking dedicated software (EchoPAC version 202, GE Healthcare, Horten, Norway) to estimate LV global longitudinal strain (GLS). Numerized strain traces (.txt files) were exported for computational analysis.

#### 2.1.3. Cardiac Magnetic Resonance Imaging

The cMRI was performed before CRT implantation using a 3-T clinical magnetic resonance system (Ingenia, Philips Medical Systems, Best, The Netherlands) with a 32-channel cardiovascular array coil. LGE images were acquired 10–15 min after intravenous administration of 0.2 mmol/kg of gadolinium (Gadoterate meglumine, Dotarem, Guerbet, Aulnay-sous-Bois, France), using 2D breath-hold inversion-recovery and phase-sensitive inversion-recovery sequences in the short-axis plane (spoiled gradient-echo, slice thickness 8 mm, repetition time 6.1 ms, echo time 2.9 ms, flip angle 25°, inversion time adjusted to null normal myocardium, typical breath-hold 11 s). The presence and localization of myocardial scar were assessed by a trained radiologist on a 16-segment LV model and the regional LGE extent was determined qualitatively and assessed on a per-segment basis.

Concerning two ischemic LBBB patients, both inner and outer contours were manually delineated on LGE-SAX images to have a precise estimation of the fibrotic burden. Then, the fuzzy c-means method proposed by [14] was used to delineate myocardial fibrosis. This is an unsupervised method classifying the voxels in the myocardium as belonging to one of two possible classes: late-gadolinium-enhanced voxel, or non-LGE voxel. This method proved appropriate correlations with biochemical myocardial infarct (scar) quantification as well as LV function parameters [15].

### 2.2. Computational Model

**Figure 1:**
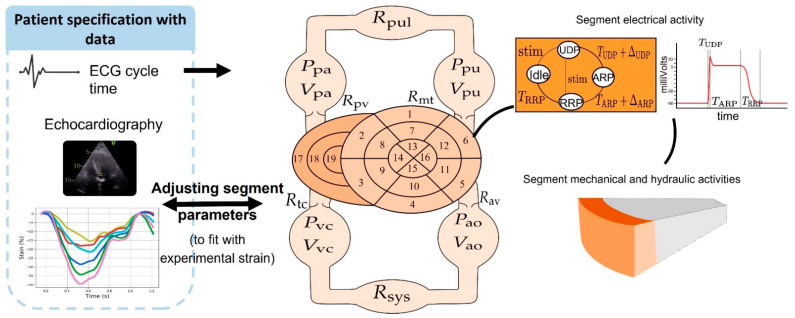
Exemplification of the exploitation of ECG and echocardiographic data obtained from patients for the construction of an electro-mechanical heart model and characteristics of the model. ((**Left**) panel)*:* ECG cycle-time and strain-derived curves obtained from each patient were compared to the outputs of the model and used to adjust the model’s outputs to increase the patient’s specification of the model. ((**Middle**) panel)*:* Closed-loop model of the cardiovascular system including a multi-segment representation of the left and right ventricles. Numbers (1 to 19) represent the different cardiac segments: (1) basal anterior; (2) basal anteroseptal; (3) basal inferoseptal; (4) basal inferior; (5) basal inferolateral; (6) basal anterolateral; (7) mid anterior; (8) mid anteroseptal; (9) mid inferoseptal; (10) mid inferior; (11) mid inferolateral; (12) mid anterolateral; (13) apical anterior; (14) apical septal; (15) apical inferior; (16) apical lateral; (17) right ventricular basal; (18) right ventricular median; (19) right ventricular apical. The systemic (sys) and pulmonary (pul) circulations include: aorta (ao), vena cava (vc), pulmonary artery (pa), and pulmonary veins (pu). ((**Right**) panel): State diagram of the cellular automaton that shows the correspondence of the transition parameters with the myocardial action potential dynamic.

The computational model included four components: (1) cardiac electrical system, (2) right and left atria, (3) multi-segment representation of the right and the left ventricles, and (4) systemic and pulmonary circulations. The model was adapted from previous works of our team [11,12,16,17,18]. Model parameters were adjusted manually to fit clinical data obtained from patients (Figure 1). To experimentally reproduce the lateral and septal strain traces of representative ischemic and non-ischemic patients with typical LBBB, we varied the value of one model parameter at a time (electro-mechanical driving, contractility, and depolarization delay) until we were able to simulate the pattern of electro-mechanical activation observed in patients.

#### 2.2.1. Electrical Activity

The cardiac electrical activity was represented by a set of interconnected cellular automata, adapted from [11,19]. Each automaton was associated with the main electrophysiological activation periods: slow diastolic depolarization (SDD), upstroke depolarization period (UDP), absolute refractory period (ARP), and relative refractory period (RRP). Each automaton cycled between these four stages/phases, sending out a stimulation signal to its neighbors when the UDP ended. The model consisted of 26 automata: the sinoatrial node (NSA), right and left atria (RA and LA), the atrioventricular node (NAV), upper bundle of His (UH), right and left bundle branches (RBB and LBB), 3 segments of right ventricle (RV) and 16 segments of left ventricle (LV). Each automaton created an electrical signal based on a template and tuned by the activation period timings. These electrical signals were gathered to create an electrocardiogram signal and access to each activation delay of the LV segments.

#### 2.2.2. Right and Left Atria

The right and left atrial pressures were defined as linear functions of instantaneous volumes and elastances representing the elastic properties of the atrial wall. This linear relation was driven by a Gaussian function that cycles between atrial diastole and systole influenced by each atria activation time, established from the stimulation of the atrial automata [16,20].

#### 2.2.3. Multi-Segment Representation of the Right and the Left Ventricles

The multi-segment model was adapted from previous work of our team [11,12]. The left ventricle wall was divided into 16 segments according to the standardized segmentation of the AHA [21]. The right ventricle wall was divided into three layers: base, medium, and apex. The left ventricular basal and medium walls were separated into six components: anterior, anteroseptal, inferoseptal, inferior, inferolateral, and anterolateral walls. The apical layer was divided into four components: anterior, septal, inferior, and lateral.

The cardiac mechanical activity was represented by active and passive components. The active tension is related with the myofiber contractility and muscle kinetic properties, and the passive tension depends on the power conservation law between LV pressure and wall tension. Longitudinal strain was calculated from current and reference fibers’ length. Each segment was activated by an electro-mechanical driving function triggered by the corresponding LV and RV automaton. The hydraulic behavior of the blood volume in contact with the wall segment was represented by its inertial and resistive effects. Ventricular flow accounted for the contribution of each segment and intra-ventricular cavity flow. Septal segment pressures depended directly on the pressure gradient across the septum.

#### 2.2.4. Systemic and Pulmonary Circulations

The cardiovascular system model integrated the right and left atria and ventricles, the cardiac valves, the pulmonary arteries, capillaries and veins, and the systemic arteries and veins [16,22]. Concerning each cavity, the pressure was related to volume using a linear relationship defined by an elastance. Then, blood flow between two chambers was calculated from the pressure gradient between the chambers and the corresponding resistance. The cardiac valves, located at the inlet and outlet of the ventricles, were represented as perfect diodes.

Figure 1 shows how ECG cycle time and strain curves obtained at transthoracic echocardiography were used to improve the patient specification of the model.

## 3. Results

### 3.1. Baseline Model Simulations

**Figure 2:**
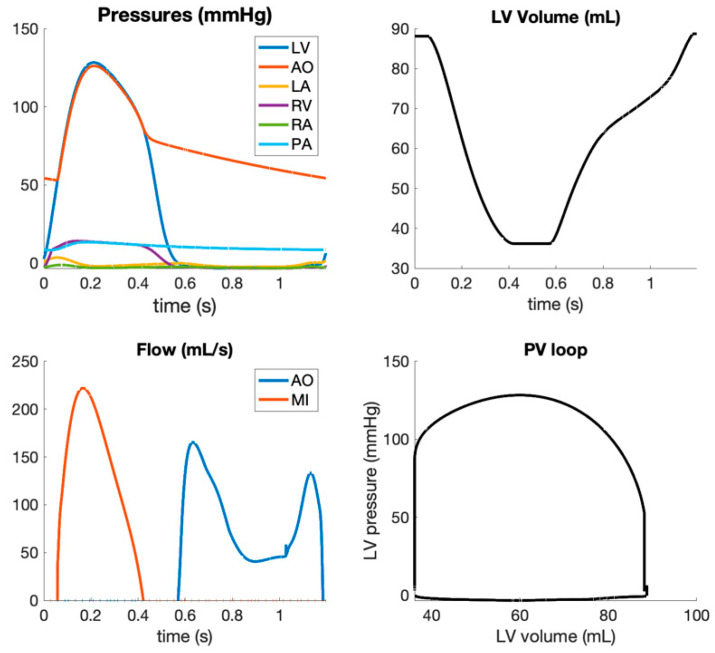
Hemodynamic simulations in baseline conditions.

Figure 2 illustrates the simulation results from the proposed computational model at baseline. Systolic LV pressure was equal to 130 mmHg, the aortic pressure varied between 60 and 130 mmHg, and LV volume varied between 40 and 90 mL; simulated aortic and mitral flows were like those observed in healthy subjects.

**Figure 3:**
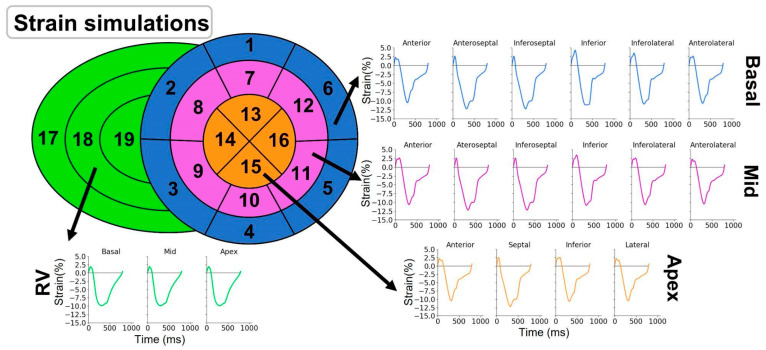
Strain simulations in baseline conditions. The presented strain curves correspond to the longitudinal deformation of each of the 16 left ventricular segments and 3 right ventricular segments during one cardiac cycle obtained after simulation of a healthy patient.

Figure 3 illustrates the strain signals obtained at baseline. Strain traces presented similar morphologies and timing across the 16 LV segments, due to the absence of LV mechanical dyssynchrony and the presence of a preserved, homogeneous LV contractility.

### 3.2. LBBB Strain Patterns

**Figure 4:**
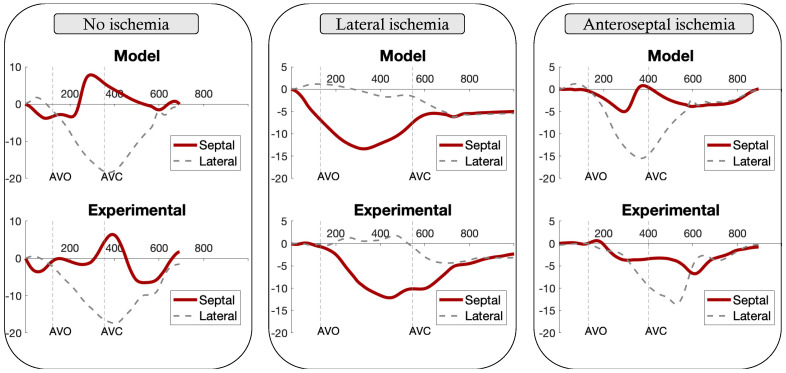
LBBB strain patterns obtained from simulated and clinical data for septal and lateral walls.

Figure 4 illustrates the experimental and model-based strain traces obtained in the septal and lateral wall for patients with non-ischemic cardiomyopathy, as well as lateral and anterior ischemia. In the case of non-ischemic LBBB, the model was able to simulate a typical septo-to-lateral activation pattern, which is characterized by a pre-ejection contraction of the septal wall followed by an immediate re-lengthening (septal rebound stretch) of the wall. In the case of lateral scar, the LV activation pattern simulated by the model was quite different and characterized by a significant reduction in lateral wall strain, loss of the septal rebound stretch, and a marked increase in septal deformation. Finally, the model simulated LV activation in the case of LBBB and anteroseptal scar. In this case, the septal deformation pattern was still characterized by an early septal shortening followed by rebound stretch. The root-mean-square errors (RMSEs) calculated between estimated and observed strain curves of the septal and lateral walls were equal to (i) 3.99% and 1.86% for the non-ischemic patient, (ii) 3.39% and 1.86% for the lateral ischemia, and (iii) 1.72% and 4.42% for the anteroseptal ischemia.

The septal wall curve was obtained as the average of the five strain curves of septal segments (apical septum, mid inferoseptum, basal inferoseptum, mid anteroseptum, and basal anteroseptum).

The lateral wall curve was obtained as the average of the five strain curves of the lateral segments (apical lateral, mid inferolateral, basal inferolateral, mid anterolateral, and basal anterolateral).

**Figure 5:**
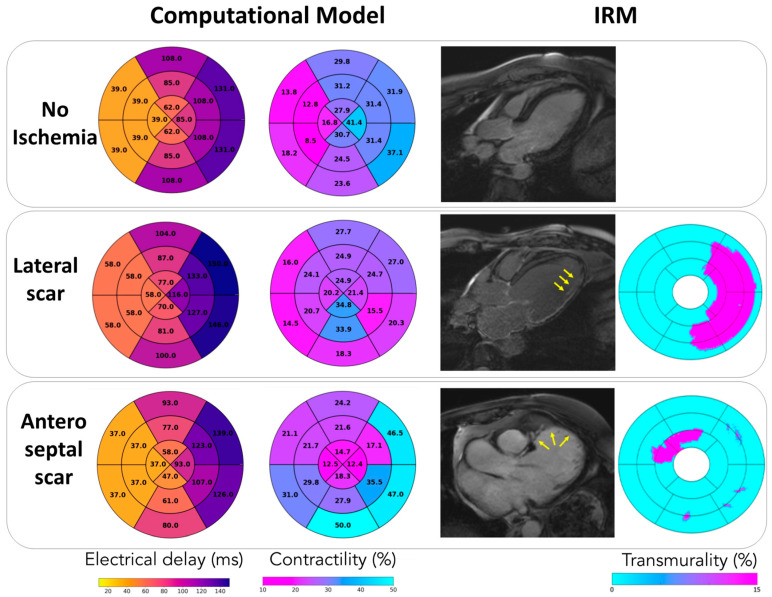
Contractility levels (%), electrical delays (ms) estimated with the model-based approach, and transmurality degree (%) in ischemic patients obtained by cMRI.

Figure 5 describes how the model was able to reproduce the LV electrical delay and localized alteration in contractility. In all cases, we observed a septal-to-lateral wall delay typical of LBBB (lower panel). Furthermore, the model was able to reproduce regional modifications in LV contractility which are due to the LBBB, but also to local scarring. In the case of isolated LBBB, we observed increased contractility of the lateral wall compared to the septum. In the case of lateral scar, we observed a significant impairment in lateral contractility. In the case of anteroseptal scar, a higher reduction in contractility was observed in the septal and apical segments. As depicted in Figure 5, reduced contractility in ischemic patients corresponded to the areas of transmural distribution of late gadolinium enhancement observed with cMRI (Figure 5). A higher percentage of transmurality translates into larger fibrotic areas, which are associated with low contractility. Therefore, regional contractility levels allow distinction between ischemic and non-ischemic cases, where reduced contractility could be associated with damaged tissues.

## 4. Discussion

In this paper we propose a model-based approach which replicates the intrinsic electromechanical properties of the myocardium observed in three real-life patients with typical LBBB undergoing CRT. Our model is also able to reproduce local alteration in LV contractility, which corresponds exactly to the areas of myocardial scar observed with cMRI.

These results were obtained by adjusting parameters such as the electrical periods of some automata or the active and passive parameters of the electro-mechanical driving function in order to simulate the LV deformation curves observed in patients with LBBB. In the case of ischemic patients, we varied the active and passive parameters of the electro-mechanical driving function to fit the experimental strain curves. At the end, we noticed that the ischemic segments also presented reduced contractility, as expressed by the smaller active parameters.

### 4.1. Strain Patterns and Scar Localization in LBBB

The presence of abnormal LV contraction patterns in patients with LBBB has been largely described and referred to as septal flash and apical rocking [23]. The visual identification of septal flash and apical rocking has been associated with a better response to CRT and better survival in CRT recipients [24]. Nevertheless, the visual identification of these contraction patterns is largely influenced by the experience of the echo-reader and might be associated with poor reproducibility. Moreover, in recent years, some pathophysiological studies have shown that these specific contraction patterns are related to both the electrical delay due to the LBBB and specific LV mechanical properties [25,26]. Namely, the presence of the septal rebound stretch, which is a known predictor of CRT response [27,28], is influenced by the presence of lateral ischemia, which abolishes the septal rebound stretch and increases septal contractility. On the other hand, anteroseptal ischemia is associated with the persistence of septal rebound stretch and a decrease in septal contractility [25,29]. Our computational model was able to disclose the characteristics of the LV deformation patterns observed in patients with typical LBBB of both ischemic and non-ischemic etiology.

Another important property of our model is its capability to reproduce LV contractility. The area of reduced contractility in ischemic patients corresponded to the localization of LV scar in patients with previous myocardial infarction. This has pivotal importance because it is well known that the localization of scar in the lateral wall can be associated with a lower rate of CRT response because of the inefficient stimulation of an ischemic territory [30]. On the other hand, Aalen et al. have recently shown that the assessment of septal viability is also important because septal recruitment through CRT delivery significantly contributes to LV reverse remodeling after CRT [31]. This seems particularly important because some patients with septal scar might have a mechanical activation pattern similar to those observed in the case of non-ischemic LBBB, which might complicate the identification of CRT responders [32].

### 4.2. Modelling and Personalized Medicine

In our study, all patients had typical LBBB of similar duration and had similar LVEF, so they were all good CRT candidates according to current recommendations. Nevertheless, the proposed model-based approach was able to reproduce the electro-mechanical delay typical of each patient and simulate regional alterations in LV contractility by the simple analysis of ECG and strain traces. The corresponding local alteration in LV performance largely corresponded with the area of scar identified by cMRI in patients with ischemic cardiomyopathy. This process might be of use in the pre-implantation phase, to aid interpretation of the pathophysiological substrate associated with the development of LBBB and potentially improve the identification of CRT responders. Therefore, the application of a model-based approach could provide additional information on the regional electrical and mechanical function of the LV and can help to disclose the intrinsic complexity of LV mechanics in CRT candidates, representing a step forward in the development of personalized LV modelling in the field of CRT.

Although previous models [7,8] successfully represented a patient diversity in LV dyssynchrony and proposed keys to understand CRT response [33], our approach provides interesting advantages and originalities. This model proposes a sub-model of the right and left ventricle, which allows not only the analysis of septal and lateral strain curves but also a complete analysis of all the ventricular regions. Contrary to the models based on the finite element method, our approach requires low computational resources, facilitating personalized therapy. Therefore, the level of detail of the model facilitates translation to clinical practice. More interestingly, our approach seems able to simulate the electrical and mechanical properties observed in patients with both ischemic and non-ischemic LBBB, in a unique and reliable way. This might improve the interpretation of diagnostic measures, provide interesting insight to the comprehension of the pathophysiological mechanisms associated with LBBB, and provide an alternative to multimodality assessment in CRT candidates.

### 4.3. Limitations and Perspectives

The main aims of computer modelling in the field of CRT are to (1) ease the comprehension of the pathophysiological mechanisms related to LBBB, (2) identify the pathophysiological substrates that are associated with CRT response, and (3) eventually help in the planification of therapy. The ideal model will be able to fully reproduce the LV electromechanical properties observed in a CRT candidate and correctly simulate the effect of CRT delivery before its effective release.

According to this goal, the main limitation of our model is the lack of parameter identification, which prevents us from proposing a patient-specific model, as developed in previous work of our team [11].

Moreover, the development of computational models for biological structures should always balance the computational costs that are necessary to obtain a detailed model with the necessary simplification process. This is also evident in our work because the simplification and assumptions made to propose a computational model for LBBB imply several limitations. At the tissue level, the segmental modelling of the ventricles induces a less-accurate representation of the electrical and mechanical behaviors such as fiber torsion or a complete mechanical continuity. Despite these simplifications, we were able to reproduce distinct strain patterns of very different patients. To consider a clinical application for our model, we should develop an automatic and robust identification process. As such, the next step of our research should be parameter identification and the validation of our results in a larger clinical dataset. After this phase, the information provided by the model will be investigated to propose keys to better understand patients’ profiles and support the planification of therapy by a patient-specific characterization.

## 5. Conclusions

The present computational model of typical LBBB is tailored to patients’ characteristics and can simulate LV electrical, mechanical, and contractility patterns encountered in real-life patients. This might provide significant help in the interpretation of septal contraction patterns and facilitate the identification of patient’s characteristics that are associated with CRT efficacy.

## Figures and Tables

**Table 1:** Main patient characteristics.

	Age (years)	Etiology	QRS Width (ms)	LVEF (%)
Patient 1	77	Non-ischemic cardiomyopathy	136	33
Patient 2	80	Ischemic cardiomyopathy—lateral scar	157	24
Patient 3	82	Ischemic cardiomyopathy—antero-septal scar	152	30

LVEF, left ventricular ejection fraction.

## Data Availability

Anonymized strain traces are available if requested.
